# New therapeutic approaches to HGPS based on progerin inhibition

**DOI:** 10.1186/1750-1172-10-S2-O8

**Published:** 2015-11-11

**Authors:** Camilla Pellegrini

**Affiliations:** 1 CNR Institute for Molecular Genetics, Unit of Bologna, Bologna, Italy, Rizzoli Orthopedic Institute, Laboratory of Musculoskeletal Cell Biology, Bologna, Italy

## 

Hutchinson-Gilford Progeria Syndrome (HGPS) is caused by a *de novo* heterozygous mutation on *LMNA* gene that leads to accumulation of progerin, a mutant form of prelamin A. HGPS skin fibroblasts are characterized by multiple nuclear defects: nuclear shape abnormalities chromatin structure alterations, increased DNA damage and cell cycle alterations.

Retinoic acid may modulate *LMNA* gene transcription, due to the presence of a retinoic acid responsive element (L-RARE) in the *LMNA* promoter. Based on this knowledge, we investigated if all trans retinoic acid (ATRA) could lower progerin levels in HGPS fibroblasts. We also evaluated the effects of a combined treatment with rapamycin, a drug known to promote autophagy and reduce both farnesylated prelamin A and progerin amount.

We demonstrate a surprising effect of ATRA to repress Lamin A/C gene transcription and we show that the combined treatment with ATRA and rapamycin has a synergistic effect: it dramatically lowers progerin levels, restores both heterochromatin organization and nuclear shape, reduces DNA damage markers and improves cell viability. These promising results could open the way to a new therapeutic approach for HGPS.

